# A Molecular Imprinted Polymer as a Flow-Through Optical Sensor for Oxazepam

**DOI:** 10.1155/2018/6302609

**Published:** 2018-04-04

**Authors:** Roberta G. Machicote, Marcela A. Castillo, Maria E. Pacheco, Liliana Bruzzone

**Affiliations:** ^1^División Química Analítica, Departamento de Química, Facultad de Ciencias Exactas, Universidad Nacional de La Plata, Calle 47, Esq. 115, 1900 La Plata, Argentina; ^2^Laboratorio de Investigación y Desarrollo de Métodos Analíticos (LIDMA), Facultad de Ciencias Exactas, Universidad Nacional de La Plata, Calle 47, Esq. 115, 1900 La Plata, Argentina

## Abstract

A flow-through optosensing system for oxazepam recognition with fluorescence detection was performed by means of a molecular imprinted polymer based on its acid hydrolysis product, 2-amino-5-chlorobenzophenone. The synthesis was conducted via a noncovalent imprinting methodology, using methacrylic acid as a functional monomer and ethylene glycol dimethacrylate as a cross-linking agent. Hydrolysis (types and concentration of acids), polymer retention capacity, binding properties, and elution (selectivity and reversibility) conditions were optimized. The selected molecular imprinted polymer had a molar ratio composition of 1 : 6 : 45 (template : functional monomer : cross-linker). The proposed method was applied to the determination of oxazepam in a pharmaceutical formulation. External standard calibration, standard additions calibration, and Youden's calibration were carried out in order to evaluate constant and proportional errors due to the matrix. The developed metabolite-based recognition system for benzodiazepines is an innovative procedure that could be followed in routine and quality control assays.

## 1. Introduction

Oxazepam (OXA), 7-chloro-l,3-dihydro-3-hydroxy-5-phenyl-2H-1,4-benzodiazepin-2-one, is a therapeutically short-to-intermediate-acting benzodiazepine used to produce sedation, induce sleep, relieve anxiety, and muscle spasms [1]. Due to its sedative properties, it has a high potential for abuse specially when it is combined with other depressants such as alcohol or opiates [2]. Urine has been the most widely used biological matrix for the determination of benzodiazepines and metabolites in drug abuse testing and toxicology [3].

Several analytical methods have been proposed for OXA determination: most of them use chromatographic techniques and others use fluorometry and capillary electrophoresis; also, some electrochemical sensors have been developed [4–13]. Since benzodiazepine metabolites are converted to benzophenone, a sensitive determination method could be performed using the hydrolyzed product [5, 14].

Molecularly imprinted polymers (MIPs) are synthetic materials that act as artificial biomimetic receivers capable to recognize and specifically interact with the analyte used as a template in the polymerization process. The specificity and selectivity gained with MIPs make them suitable as recognition elements for chemical and biochemical sensors [15–28].

In this work, a rapid, simple, and low-cost analytical method is proposed for the indirect determination of OXA in pharmaceutical formulations based on the development of an optical sensor for its acid hydrolysis product, 2-amino-5-chlorobenzophenone (BZF). A MIP has been synthesized via the noncovalent imprinting methodology as a recognition element in a flow-through optosensing system with fluorescence detection.

## 2. Materials and Methods

### 2.1. Materials

Methacrylic acid (MAA) was purchased from Fluka (Buchs, Switzerland), and ethylene glycol dimethacrylate (EGDMA) and α,α'-azoisobutyronitrile (AIBN) were acquired from Aldrich (Milwaukee, WI, USA). They were purified as described in a previous work [29].

All other chemicals were of analytical grade and used as received. Oxazepam (OXA) was kindly supplied by a pharmaceutical store. 2-amino-5-chlorobenzophenone (BZF) (purity 98%) was acquired from Aldrich (Milwaukee, WI, USA). A pharmaceutical formulation (tablets) containing OXA was purchased from a local store. Double-distilled water was used.

Stock solutions of OXA and BZF were prepared in adequate solvents as needed for the planned assays.

### 2.2. Apparatus

All recordings of fluorescence spectra and fluorimetric measurements were carried out on a PerkinElmer LS-50B luminescence spectrometer (Beaconsfield, England) equipped with a pulsed xenon lamp (half peak height <10 *μ*s, 60 Hz), an R928 photomultiplier tube, and a computer working with FL Winlab software.

The optosensing manifold used for the luminescence measurements was similar to the one employed in a previous work [29]. A 25 *μ*L, 1.5 mm path length, conventional flow-through quartz cell (Hellma, model 176.052-QS, Mullheim, Germany) was loaded with the synthesized MIP [29]. The as-packed active phase was used with satisfactory readings for several months. (All the experiments were carried out without changing the active phase from the flow cell.)

A four channel Gilson's Minipuls 3 peristaltic pump (Villiers-le-Bel, France) fitted with organic solvent resistant Tygon tubing was used to generate the flowing streams. A Rheodyne 5020 rotatory valve (Rohnert Park, CA, USA) provided with a 500 *μ*L loop was employed for sample introduction. PTFE tubes (0.8 mm internal diameter) were used throughout the flow system.

Gas chromatographic analysis (GC) were performed with a Shimadzu GC-2014 (Japan) provided with an AOC-20i automatic injector and FID detector working with a Restek RTx-65TG capillary column.

Absorption spectra were recorded on a Shimadzu UV-240 (Japan) recording spectrophotometer, equipped with a 1.0 cm path length quartz cell. Derivative absorption spectra were performed applying simple mathematical calculations.

### 2.3. Oxazepam Hydrolysis

As temperature alone does not produce the OXA hydrolysis, aliquots of OXA working solutions were initially mixed with 0.1 M acid solutions (sulphuric, acetic, and hydrochloric acids) in screw-capped tubes and hydrolyzed at 100°C for 60 minutes to form BZF. After cooling, absorption spectra were registered and their first-order derivative spectra were obtained.

### 2.4. MIP/NIP Synthesis

A molecular imprinted polymer (MIP) with BZF was synthesized according to a standard method previously studied in our research group [29]. As BZF has an amino group, methacrylic acid (MAA) has been selected as a functional monomer. In a screw-capped tube, a total of 21 mg of BZF (0.09 mmol), template, were dissolved in 2 mL of chloroform, porogen, by sonication for 15 minutes. Afterwards, 531 *μ*L (2.8 mmol) of EGDMA, a cross-linking agent, and 32 *μ*L (0.37 mmol) of MAA were added and shaken in a Vortex during another 15 minutes. With the tube immersed in an ice bath, 3 mg (0.019 mmol) of AIBN, a radical initiator, was added and sonicated for 5 minutes. The mixture was degassed by bubbling *N*_2_ for 10 minutes. The tube was heated at 60°C for 16 hours. The polymer was removed from the tube, ground in an agatha mortar, and sieved to an average particle size of 70–100 mesh. The particles were washed in the Soxhlet extractor with acetic acid : methanol (10% v/v) until BZF could no longer be detected by UV absorption in the washing solution. Finally, the particles were washed with methanol and dried at 40°C during 24 hours. A nonimprinted polymer (NIP) was synthesized using the same procedure without the addition of BZF.

### 2.5. Binding Assays

Binding assays were performed contacting each MIP/NIP during 48 hours with BZF chloroformic standard solutions. After this time, the mixture was centrifuged and the supernatant was analyzed by gas chromatography (GC). A calibration graph with standard BZF solutions was prepared in order to quantify BZF concentration. The BZF concentration bonded to the polymer was calculated by subtracting the BZF concentration in the supernatant to the initial one.

### 2.6. Oxazepam Determination

The proposed method was applied to the determination of OXA in a pharmaceutical formulation (Pausafren T, 15 mg OXA, tablets). Five tablets were accurately weighed, powdered in an agatha mortar, and homogenized. An average mass of 1.150 g per tablet was calculated.

## 3. Results and Discussion

### 3.1. Oxazepam Hydrolysis

**Figure 1:**
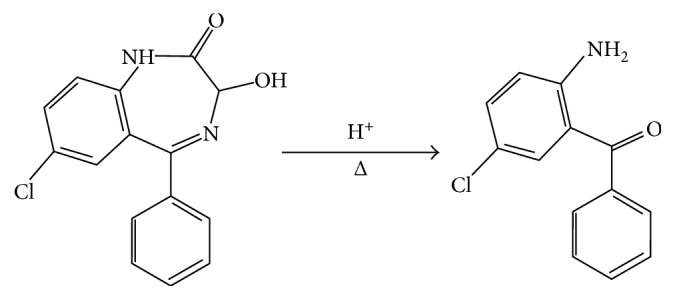
Oxazepam acid hydrolysis reaction.

The acid hydrolysis of benzodiazepines to benzophenones has been previously reported [30, 31]. The hydrolysis product of oxazepam (OXA), 2-amino-5-chlorobenzophenone (BZF), is shown in Figure 1.

**Figure 2:**
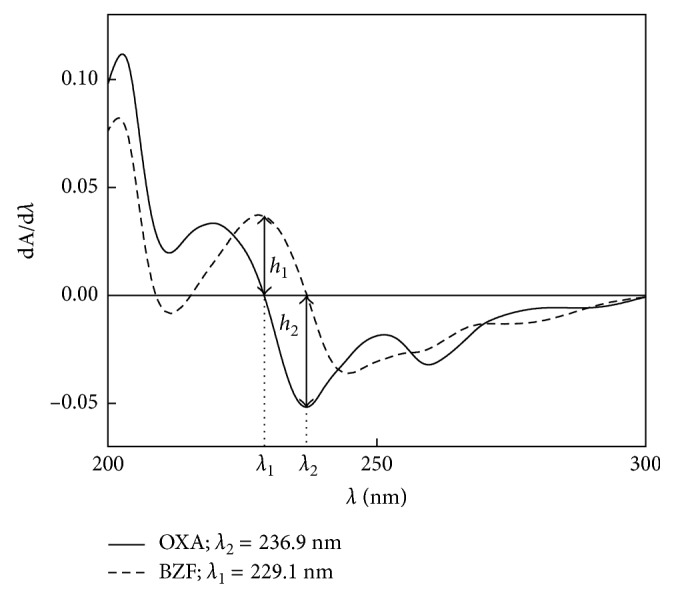
OXA and BZF first-order absorption derivative spectra.

As absorption spectra of OXA and its respective benzophenone are overlapped, it is difficult to determine the BZF concentration in the presence of OXA when a partial hydrolysis takes place. The OXA and BZF first-order derivative spectra have two wavelengths at which dA/dλ are zero: 236.9 nm and 229.1 nm (Figure 2). These wavelengths are suitable for the quantification of both species after different hydrolysis treatments: BZF at 229.1 nm and OXA at 236.9 nm.

**Figure 3:**
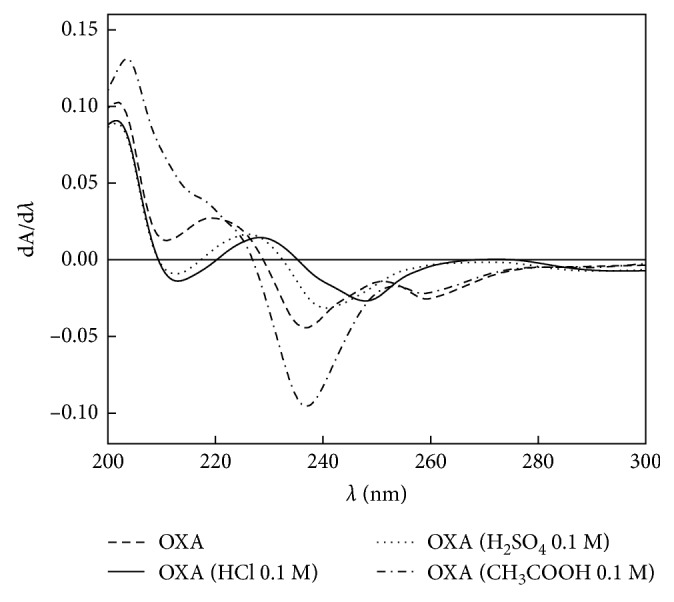
OXA first-order absorption derivative spectra after partial hydrolysis. [OXA] = 3.50 × 10^−5^ M.

**Figure 4:**
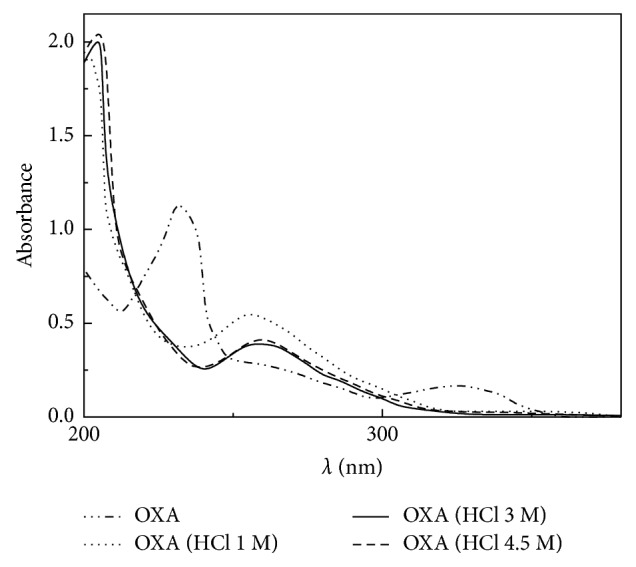
OXA first-order absorption derivative spectra after complete hydrolysis. [OXA] = 3.02 × 10^−5^ M.

Absorbances were measured at the mentioned wavelengths (Figure 3). From these values, it was deduced that the hydrolysis takes place with major efficiency in the presence of hydrochloric acid (HCl). The tested concentration was inadequate to produce complete hydrolysis. Afterwards, the hydrolysis reaction was performed with a series of increasing HCl molar concentration solutions. A 3 M HCl concentration was selected as the optimum one for the complete hydrolysis (Figure 4).

### 3.2. Binding Assays

**Figure 5:**
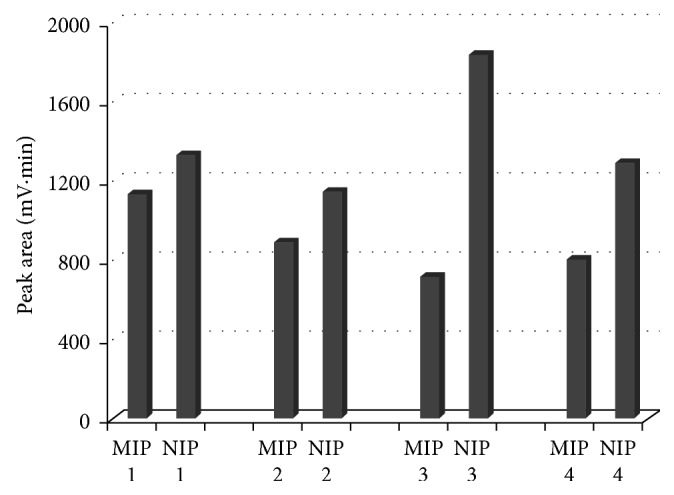
BZF peak area for each MIP/NIP after incubation time.

In order to select the best retention capacity of the MIP, a series of polymers were synthesized maintaining a 7.5 EGDMA/MAA molar ratio (Table 1). The polymers were synthesized, ground, sieved, washed, and dried as previously stated. 2 mL of a 2.52 × 10^−4^ M BZF chloroformic solution was added to 13 mg of each MIP in a capped test tube. The tubes were shaken in a Vortex and let to stand for 48 hours at room temperature. After this time, the tubes were centrifuged at 4000 rpm for 5 minutes and the solutions were GC analyzed. Results are shown in Figure 5. All MIPs retain a greater BZF quantity than the NIPs. MIP #3 shows the best retention with an optimal MAA/BZF molar ratio equal to 6.

**Figure 6:**
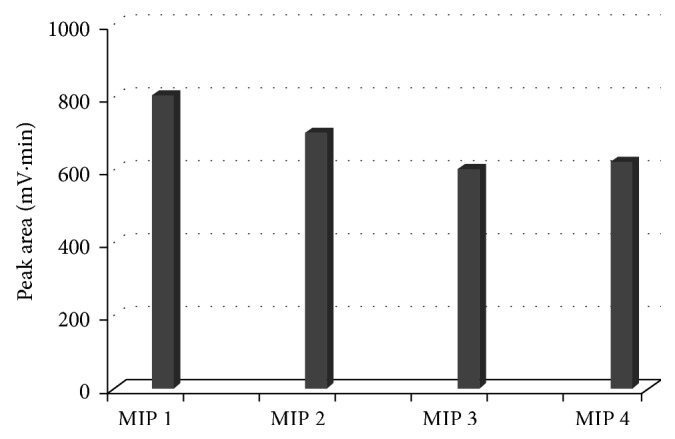
BZF peak area for each MIP after incubation time.

In order to evaluate the optimum EGDMA/MAA molar ratio, a series of polymers were synthesized maintaining a 6 MAA/BZF molar ratio (Table 2). The polymers were synthesized, ground, sieved, washed, and dried. Binding assays were performed as abovementioned. Results are shown in Figure 6. MIP #3 presents the major retention capacity with a 7.5 EGDMA/MAA molar ratio.

### 3.3. MIP/NIP Optimized Synthesis: Optosensing System Evaluation

The working polymers (MIP and NIP) were synthesized under the previously stated conditions (Section 3.2).

**Figure 7:**
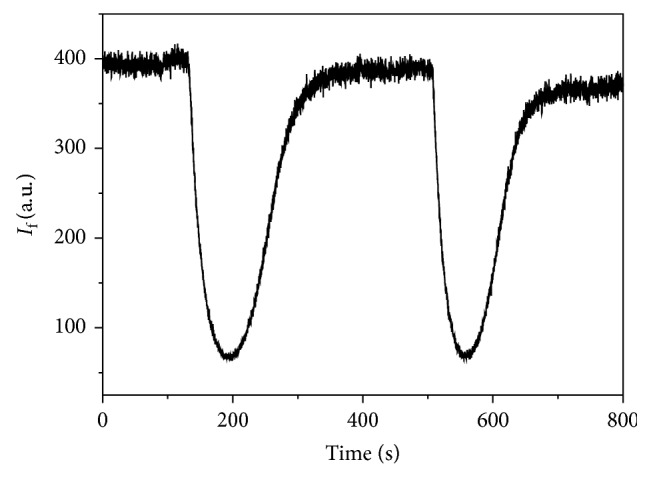
MIP response profile for a 1.28 × 10^−5^ M BZF methanolic solution.

**Figure 8:**
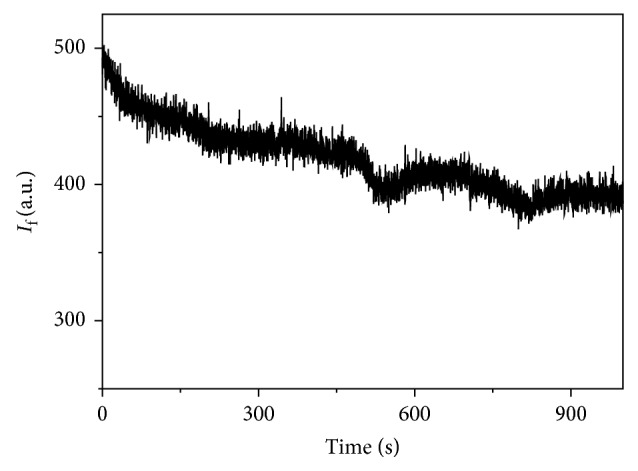
NIP response profile for a 1.28 × 10^−5^ M BZF methanolic solution.

The MIP was introduced in the flow cell of the optosensing system and was evaluated in a simple way with a methanol carrier solution containing 10% v/v acetic acid. The fluorescent signal was monitored setting the excitation and emission wavelengths at 220 nm and 336 nm, respectively, with 10 nm of excitation and emission bandwidths. A carrier flow rate of 4 mL/min was used, and a stable signal was obtained. When a 1.28 × 10^−5^ M BZF methanolic solution was injected, a negative peak was obtained returning to the baseline after a definite period of time. Certainly, the MIP recognizes and retains the BZF. The inverted peaks suggest that the interaction produces a quenching of the MIP fluorescence (Figure 7). No appreciable changes were detected when NIP was assayed (Figure 8).

**Figure 9:**
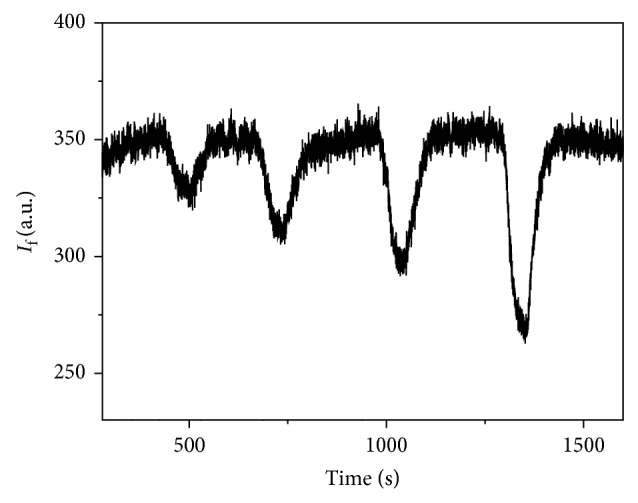
MIP response profile for different BZF methanolic solution concentrations: 1.78 × 10^−5^ M, 3.40 × 10^−5^ M, 4.98 × 10^−5^ M, and 7.12 × 10^−5^ M.

Under the conditions previously established, solutions of different BZF concentrations were injected in the system.  Figure 9 shows the diagram when different concentrations of BZF methanolic solutions were injected into the flow system using the synthesized MIP as the sensing material. The regeneration with the carrier solution is also shown.

The polarity of the carrier solvent affects the BZF binding properties. A solvent with high polarity competes with the sample molecules for binding at the recognition sites. The result is a weakness in the specific polar interaction leading BZF to go to the carrier solution. Otherwise, in nonpolar solvent, the molecule stays into the polymer. Thus, a nonpolar solvent should be used for detecting the quenching of the fluorescence caused by the BZF retention. A polar solvent should be then used for eluting the molecule from the polymer leaving the MIP in the unbound situation for receiving a new injection.

**Figure 10:**
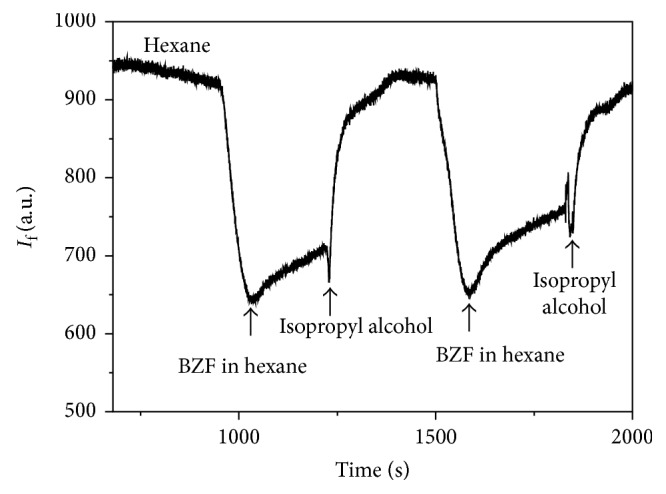
Fluorescence response profile for BZF removal/rebinding in a MIP.

**Figure 11:**
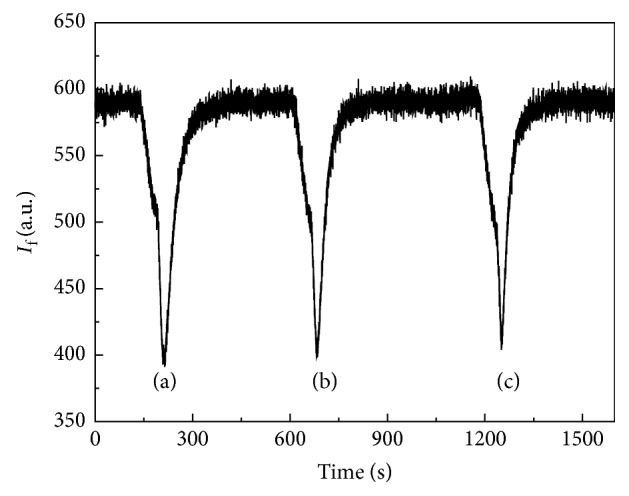
MIP response profile for BZF with different hexane/isopropyl alcohol mixtures as a carrier: (a) 90 : 10 (v/v), (b) 70 : 30 (v/v), and (c) 30 : 70 (v/v).

A first attempt was made with hexane as a nonpolar carrier solvent injecting a 3.40 × 10^−5^ M BZF solution in hexane followed by an isopropyl alcohol injection. Experimental working parameters were optimized; excitation and emission wavelengths were set at 410 and 515 nm, respectively, with 10 nm and 5 nm corresponding bandwidths. This situation is shown in Figure 10. As can be seen, the change of solvents requires a too long time to restore the baseline leading to a nonsymmetrical peak and making the study unpractical. The next step was searching for a solvent that could let the BZF interact with the polymer (selectivity) and simultaneously be capable of reaching the initial conditions without changing it (reversibility). As hexane and isopropyl alcohol are miscible, different proportions of both were assayed. A 3.40 × 10^−5^ M BZF solution in hexane was injected in 90 : 10, 70 : 30, and 30 : 70 (v/v) hexane/isopropyl alcohol mixtures. The obtained results showed that the peaks widths were diminished when the content of isopropyl alcohol was increased (Figure 11). On the other hand, the baseline was quickly restored, but the intensity was diminished. A 90 : 10 (v/v) ratio of hexane/isopropyl alcohol was selected for further studies. The carrier flow rate showed a similar behavior: an increase of its velocity produced a narrow peak width with low intensity on account of the lesser retention. A carrier flow rate of 5 mL/min was chosen.

### 3.4. Oxazepam Determination

Two kinds of calibration graphs were performed: (i) with BZF standards and (ii) with a solution of previously hydrolyzed OXA.

- Solutions of BZF were prepared in 3 M HCl. 3 mL of each one were extracted with the same volume of hexane.
- Solutions prepared with the obtained product after OXA acid hydrolysis were treated in the same way as in (i).

The organic phases were separated and injected in the flow system, and the peak heights were measured. Calibration graphs were obtained for the two cases and were analyzed using a *t*-test in order to compare both slopes [32]. It could be concluded that there is not any significant difference between the two calibration graphs. Therefore, calibration with BZF standards may be utilized for OXA quantification in a real sample (previous acid hydrolysis).

External standard and standard additions calibration graphs were carried out.

- External standard calibration graph: adequate aliquots of 1.24 × 10^−3^ M BZF stock solution were transferred to a series of 10.00 mL calibrated flasks attaining a final concentration range of 1.25 × 10^−5^ M–7.50 × 10^−5^ M after dilution with 3 M HCl.
- Standard additions calibration graph: portions of 0.300 g of the sample were spiked with different amounts of OXA, diluted to 10.00 mL with 3 M HCl, and hydrolyzed; one milliliter of the so-obtained solution was diluted to 10.00 mL with 3 M HCl resulting in a mass range of 0–0.0125 g.

In both cases, extraction with hexane was performed and solutions were injected in the flow system. The slopes of both calibration graphs were compared by means of a *t*-test [32]. The results indicated the presence of an analyte/matrix interaction effect.

As matrix effects were found, the quantitative evaluation of the analyte was carried out by the standard additions and Youden calibration [33].

- Standard additions calibration graph was obtained by weighing five equal portions of the ground tablets, spiked with different amounts of OXA and individually used to prepare the solutions required for the flow injection system.
- Youden's calibration graph was obtained by weighing four different amounts of the ground tablets in the range of 0.25–0.85 g. Each of them was individually used to prepare the solutions required for the flow injection system.

The results of both calibration graphs are shown in Table 3.

Considering the mass of each tablet and the amount used to obtain the standard additions graph, the OXA content was calculated from the ratio of the difference between the ordinate intercepts of the standard additions and the Youden plots to the slope of the standard additions calibration graph. A value of 15.1 ± 0.9 mg OXA per tablet was obtained, in agreement with the content provided by the manufacturer (15 mg OXA per tablet). Matrix effects may be possible due to the presence of *β*-alanine in the sample. *β*-alanine has an amino group that can interact with MAA and thus interfere with the BZF recognition. Taking into account that the product contains 500 mg of *β*-alanine per tablet, the obtained result is certainly acceptable. The extraction with hexane may be another cause for the matrix effect.  Table 4 summarizes the analytical figures of merit of the proposed methodology.

## 4. Conclusions

A novel optosensor for oxazepam has been developed based on its acid hydrolysis product, 2-amino-5-chlorobenzophenone. The novel use of the metabolite is beneficial in terms of the unnecessary handling of benzodiazepines. The proposed analytical methodology was applied for oxazepam determination in a pharmaceutical formulation, avoiding matrix effects by means of the Youden calibration method. The developed fluorescence flow-through optosensing system has wider impacts on other domains in society other than academics as it is suitable for routine assays and quality control in pharmaceutical industries and drug abuse divisions as an alternative for chromatographic methodologies. It opens up possibilities for knowledge to be used in unexpected, creative, and innovative ways beyond normal professional research.

## Figures and Tables

**Table 1:** Polymers synthesized maintaining a 7.5 EGDMA/MAA molar ratio.

MIP	[MAA]/[BZF]	(mmol)		
		BZF	MAA	EGDMA
1	2	0.043	0.086	0.647
2	4	0.043	0.172	1.290
3	6	0.043	0.258	1.935
4	8	0.043	0.344	2.580

**Table 2:** Polymers synthesized maintaining a 6 MAA/BZF molar ratio.

MIP	[EGDMA]/[MAA]	(mmol)		
		BZF	MAA	EGDMA
1	2.5	0.043	0.258	0.645
2	5	0.043	0.258	1.290
3	7.5	0.043	0.258	1.935
4	10	0.043	0.258	2.580

**Table 3:** Results of the least-squares regression analysis.

	Standard additions	Youden's calibration
*n*	10	8
Intercept	23.6	7.2
Slope	4165 g^−1^	49 g^−1^
*s*_a_	0.9	0.7
*s*_b_	116 g^−1^	1 g^−1^
*s*_*y*/*x*_	20.52	3.22
*r*	0.9969	0.9984

*n*: number of calibration data points; *s*_a_: standard deviation of the intercept; *s*_b_: standard deviation of the slope; *s*_*y*/*x*_: standard deviation of the regression; r: correlation coefficient.

**Table 4:** Figures of merit.

SEN^†^	0.42 mL·*μ*g^−1^
*γ*	0.26 mL·*μ*g^−1^
LOD	11.2 *μ*g·mL^−1^
LOQ	34.2 *μ*g·mL^−1^
Dynamic range	11.2–123.3 *μ*g·mL^−1^
Linear range	34.2–123.3 *μ*g·mL^−1^

^†^SEN: calibration sensitivity; *γ*: analytical sensitivity; LOD: limit of detection; LOQ: limit of quantification; LOD = 3.28 × *s*_0_; LOQ = 10 × *s*_0_; $s_{0}=\left(s_{y/x}/b\right)\sqrt{\left(1/\sum_{i}n_{i}\right)+\left(1/m\right)+\left({\bar{x}}^{2}/\sum_{i}{\left(x_{i}-\bar{x}\right)}^{2}\right)}$.

## References

[1] Page C., Michael C., Sutter M., Walker M., Hoffman B. B. (2002). *Integrated Pharmacology*.

[2] Brands B., Sproule B., Marshman J. (1998). *Drugs & Drug Abuse*.

[3] Hawks R. L., Nora Chiang C. (1986). *Urine Testing for Drugs of Abuse*.

[4] Zapardiel A., Perez Lopez J. A., Bermejo E., Hernandez L., Chicharro M. (1991). Voltammetric studies on the interactions between camazepam metabolic series and human serum albumin. Determination of oxazepam using adsorptive stripping voltammetry. *Analytica Chimica Acta*.

[5] DolejSová J., Solich P., Polydorou C. K., Koupparis M. A., Efstathiou C. E. (1999). Flow-injection fluorimetric determination of 1,4-benzodiazepines in pharmaceutical formulations after acid hydrolysis. *Journal of Pharmaceutical and Biomedical Analysis*.

[6] McClean S., O’Kane E., Hillis J., Smyth W. F. (1999). Determination of 1,4-benzodiazepines and their metabolites by capillary electrophoresis and high-performance liquid chromatography using ultraviolet and electrospray ionisation mass spectrometry. *Journal of Chromatography A*.

[7] Pham-Huy C., Villain-Pautet G., Hua H. (2002). Separation of oxazepam, lorazepam, and temazepam enantiomers by HPLC on a derivatized cyclodextrin-bonded phase: application to the determination of oxazepam in plasma. *Journal of Biochemical and Biophysical Methods*.

[8] Pirnay S., Hervé F., Bouchonnet S., Perrin B., Baud F. J., Ricordel I. (2006). Liquid chromatographic–electrospray ionization mass spectrometric quantitative analysis of buprenorphine, norbuprenorphine, nordiazepam and oxazepam in rat plasma. *Journal of Pharmaceutical and Biomedical Analysis*.

[9] Lozano-Chaves M. E., Palacios-Santander J. M., Cubillana-Aguilera L. M., Naranjo-Rodríguez I., Hidalgo-Hidalgo-de-Cisneros J. L. (2006). Modified carbon-paste electrodes as sensors for the determination of 1,4-benzodiazepines: application to the determination of diazepam and oxazepam in biological fluids. *Sensors and Actuators B: Chemical*.

[10] Gil Tejedor A. M., Fernández Hernando P., Durand Alegría J. S. (2007). A rapid fluorimetric screening method for the 1,4-benzodiazepines: determination of their metabolite oxazepam in urine. *Analytica Chimica Acta*.

[11] Bastos Borges K., Figueiredo Freire E., Martins I., Pereira Bastos de Siqueira M. E. (2009). Simultaneous determination of multibenzodiazepines by HPLC/UV: investigation of liquid–liquid and solid-phase extractions in human plasma. *Talanta*.

[12] Magrini L., Cappiello A., Famiglini G., Palma P. (2016). Microextraction by packed sorbent (MEPS)-UHPLC-UV: a simple and efficient method for the determination of five benzodiazepines in an alcoholic beverage. *Journal of Pharmaceutical and Biomedical Analysis*.

[13] Jiang F., Rao Y., Wang R. (2016). Sensitive, automatic method for the determination of diazepam and its five metabolites in human oral fluid by online solid-phase extraction and liquid chromatography with tandem mass spectrometry. *Journal of Separation Science*.

[14] Roškar R., Sollner Dolenc M. (2010). Determination of benzodiazepines in urine via benzophenone derivatives using liquid chromatography-tandem mass spectrometry. *Archives of Industrial Hygiene and Toxicology*.

[15] Wulff G. (1995). Molecular imprinting in cross-linked materials with the aid of molecular templates-a way towards artificial antibodies. *Angewandte Chemie International Edition*.

[16] Mayes A. G., Mosbach K. (1997). Molecularly imprinted polymers: useful materials for analytical chemistry?. *Trends in Analytical Chemistry*.

[17] Sellergren B. (1997). Noncovalent molecular imprinting: antibody-like molecular recognition in polymeric network materials. *Trends in Analytical Chemistry*.

[18] Al-Kindy S., Badía R., Suárez-Rodríguez J. L., Díaz-García M. E. (2000). Molecularly imprinted polymers and optical sensing applications. *Critical Reviews in Analytical Chemistry*.

[19] Sellergren B. (2001). *Molecularly Imprinted Polymers. Man-Made Mimics of Antibodies and their Applications in Analytical Chemistry*.

[20] Kandimalla V. B., Ju H. (2004). Molecular imprinting: a dynamic technique for diverse applications in analytical chemistry. *Analytical and Bioanalytical Chemistry*.

[21] Karim K., Breton F., Rouillon R., Piletska E. V., Guerreiro A., Chianella I. (2005). How to find effective functional monomers for effective molecularly imprinted polymers?. *Advanced Drug Delivery Reviews*.

[22] Kloskowski A., Pilarczyk M., Przyjazny A., Namiesnik J. (2009). Progress in development of molecularly imprinted polymers as sorbents for sample preparation. *Critical Reviews in Analytical Chemistry*.

[23] Li J., Li Y., Zhang Y., Wei G. (2012). Highly sensitive molecularly imprinted electrochemical sensor based on the double amplification by an inorganic prussian blue catalytic polymer and the enzymatic effect of glucose oxidase. *Analytical Chemistry*.

[24] Jenkins A. L., Ellzy M. W., Buettner L. C. (2012). Molecularly imprinted polymer sensors for detection in the gas, liquid, and vapor phase. *Journal of Molecular Recognition*.

[25] Hawari H. F., Samsudin N. M., Shakaff A. Y. (2013). Highly selective molecular imprinted polymer (MIP) based sensor array using interdigitated electrode (IDE) platform for detection of mango ripeness. *Sensors and Actuators B: Chemical*.

[26] Wang X., Luo J., Yi C., Liu X. (2013). Paracetamol sensor based on molecular imprinting by photosensitive polymers. *Electroanalysis*.

[27] Li L., Liang Y., Liu Y. (2013). Designing of molecularly imprinted polymer-based potentiometric sensor for the determination of heparin. *Analytical Biochemistry*.

[28] Gurtova O., Ye L., Chmilenko F. (2013). Potentiometric propranolol-selective sensor based on molecularly imprinted polymer. *Analytical and Bioanalytical Chemistry*.

[29] Andreetta H. A., Bruzzone L. (2008). Fluorescence detection of atenolol using a molecular imprinted polymer. *Analytical Letters*.

[30] Morales-Rubio A., Julián-Ortiz J. V., Salvador A., de la Guardia M. (1994). Hydrolysis of benzodiazepines in a microwave oven and ultraviolet derivative analysis of their benzophenones. *Microchemical Journal*.

[31] Gambart D., Cárdenas S., Gallego M., Valcárcel M. (1998). An automated screening system for benzodiazepines in human urine. *Analytica Chimica Acta*.

[32] Massart D. L., Vandeginste B. G. M., Buydens L. M. C., De Jong S., Lewi P. J., Smeyers-Verbeke J. (1997). *Handbook of Chemometrics and Qualimetrics: Part A*.

[33] Castells R. C., Castillo M. A. (2000). Systematic errors: detection and correction by means of standard calibration, Youden calibration and standard additions method in conjunction with a method response model. *Analytica Chimica Acta*.

